# Pyridostigmine Protects Against Diabetic Cardiomyopathy by Regulating Vagal Activity, Gut Microbiota, and Branched-Chain Amino Acid Catabolism in Diabetic Mice 

**DOI:** 10.3389/fphar.2021.647481

**Published:** 2021-05-18

**Authors:** Yang Yang, Ming Zhao, Xi He, Qing Wu, Dong-Ling Li, Wei-Jin Zang

**Affiliations:** Department of Pharmacology, School of Basic Medical Sciences, Xi’an Jiaotong University Health Science Center, Xi’an, China

**Keywords:** diabetic cardiomyopathy, gut microbiota, branched-chain amino acids, pyridostigmine, mitochondrial dysfunction, vagal activity

## Abstract

The disruption of gut microbes is associated with diabetic cardiomyopathy, but the mechanism by which gut microbes affect cardiac damage remains unclear. We explored gut microbes and branched-chain amino acid (BCAA) metabolite catabolism in diabetic cardiomyopathy mice and investigated the cardioprotective effect of pyridostigmine. The experiments were conducted using a model of diabetic cardiomyopathy induced by a high-fat diet + streptozotocin in C57BL/6 mice. The results of high-throughput sequencing showed that diabetic cardiomyopathy mice exhibited decreased gut microbial diversity, altered abundance of the diabetes-related microbes, and increased abundance of the BCAA-producing microbes Clostridiales and Lachnospiraceae. In addition, diabetes downregulated tight junction proteins (ZO-1, occludin, and claudin-1) and increased intestinal permeability to impair the intestinal barrier. These impairments were accompanied by reduction in vagal activity that manifested as increased acetylcholinesterase levels, decreased acetylcholine levels, and heart rate variability, which eventually led to cardiac damage. Pyridostigmine enhanced vagal activity, restored gut microbiota homeostasis, decreased BCAA-producing microbe abundance, and improved the intestinal barrier to reduce circulating BCAA levels. Pyridostigmine also upregulated BCAT2 and PP2Cm and downregulated p-BCKDHA/BCKDHA and BCKDK to improve cardiac BCAA catabolism. Moreover, pyridostigmine alleviated abnormal mitochondrial structure; increased ATP production; decreased reactive oxygen species and mitochondria-related apoptosis; and attenuated cardiac dysfunction, hypertrophy, and fibrosis in diabetic cardiomyopathy mice. In conclusion, the gut microbiota, BCAA catabolism, and vagal activity were impaired in diabetic cardiomyopathy mice but were improved by pyridostigmine. These results provide novel insights for the development of a therapeutic strategy for diabetes-induced cardiac damage that targets gut microbes and BCAA catabolism.

## Introduction

According to the report released by the International Diabetes Federation in 2019, there were 463 million people with diabetes aged 20–79 years, and that number is estimated to rise to 700 million by 2045 (Saeedi et al., 2019). The chronic and systemic influences of hyperglycemia, hyperlipidemia, reactive oxygen species (ROS) overproduction, inflammatory cytokine activation, and concomitant metabolic changes associated with diabetes damage multiple organs and tissues, including the eyes, kidneys, blood vessels, nerves, and heart (Hu et al., 2017). Cardiovascular disease is recognized as a major cause of morbidity and mortality among diabetic patients. Diabetic cardiomyopathy, a major cardiovascular complication in diabetic patients, is defined as structural and functional myocardial impairment without coronary artery disease or hypertension (Cosentino et al., 2020). Although the pathophysiological mechanisms leading to diabetic cardiomyopathy are certainly multifactorial, increasing evidence suggests that abnormalities in the gut microbiota contribute substantially to the development of cardiac dysfunction in diabetes and thus to the pathogenesis of diabetic cardiomyopathy. Diabetic patients and animals exhibit significantly different gut microbiota than their nondiabetic counterparts (Wang and Jia, 2016). Gut microbiota homeostasis is disrupted in the contexts of cardiovascular diseases such as coronary heart disease, hypertension, heart failure, ventricular fibrillation, and vascular dysfunction (Lee et al., 2018). Modification of host metabolism *via* targeting the gut microbiota is a novel avenue for the prevention and treatment of diabetic encephalopathy and diabetic nephropathy (Li Y. et al., 2020). The effects may be mediated partly through metabolome components, especially branched-chain amino acids (BCAAs), and several bacterial species are associated with regulation of BCAA biosynthesis (Pedersen et al., 2016).

BCAA supplementation is often beneficial to energy expenditure; however, elevated circulating BCAA levels may be a risk factor for diabetes (Newgard et al., 2009; Ottosson et al., 2018). The serum metabolomes of insulin-resistant individuals are characterized by increased levels of BCAAs, which are correlated with strong potential of the gut microbiome for BCAA biosynthesis (Pedersen et al., 2016; Ottosson et al., 2018). In addition, high-circulating BCAA levels are accompanied by tissue-specific inactivation of BCAA-catabolizing enzymes in human and animal studies (Zhou et al., 2019). Furthermore, BCAAs augment the production of mitochondria-derived ROS with subsequent increases in oxidative damage and mitochondrial dysfunction (Jiang et al., 2021). Mitochondrial dysfunction has been identified as a relevant mechanism in cardiometabolic diseases and underlying cardiovascular risk factors, such as diabetes, hypertension, and atherosclerosis (Zhenyukh et al., 2017). From this perspective, targeting the gut microbiota to improve abnormalities in circulating BCAAs could be a pivotal strategy for improvement of cardiac function.

Recent studies have revealed that autonomic imbalance occurs frequently in humans with diabetes (Johnson and Wilson, 2018). Autonomic imbalance participates in the pathological processes of cardiovascular diseases (Lu et al., 2018). Pyridostigmine, a reversible cholinesterase inhibitor, has a quaternary carbamine group and a poor ability to cross the blood–brain barrier (Soares et al., 2004). Pyridostigmine increases acetylcholine (ACh) levels by preventing hydrolysis of ACh and enhancing the efficiency of cholinergic transmission, improving cardiac function in patients with cardiovascular diseases (Androne et al., 2003). There have been few investigations into the role of the gut microbiota in the cardioprotective effects of vagal nerve activation and its mechanism in diabetic cardiomyopathy mice. Therefore, the present study was performed to investigate the effects of pyridostigmine on the gut microbiota and BCAA catabolism in diabetic cardiomyopathy mice.

## Materials and Methods

### Animals and Experimental Models

Male C57BL/6J mice (4–6 weeks old) were supplied by the Experimental Animal Center of Xi’an Jiaotong University. The animals were maintained under standard laboratory conditions and housed in a temperature-controlled room with ad libitum access to water and food unless otherwise indicated. All experimental procedures were performed in accordance with the Guidelines for the Care and Use of Laboratory Animals (National Institutes of Health Publication, eighth edition, 2011). This study was approved by the Ethics Committee of Xi’an Jiaotong University.

**FIGURE 1:**
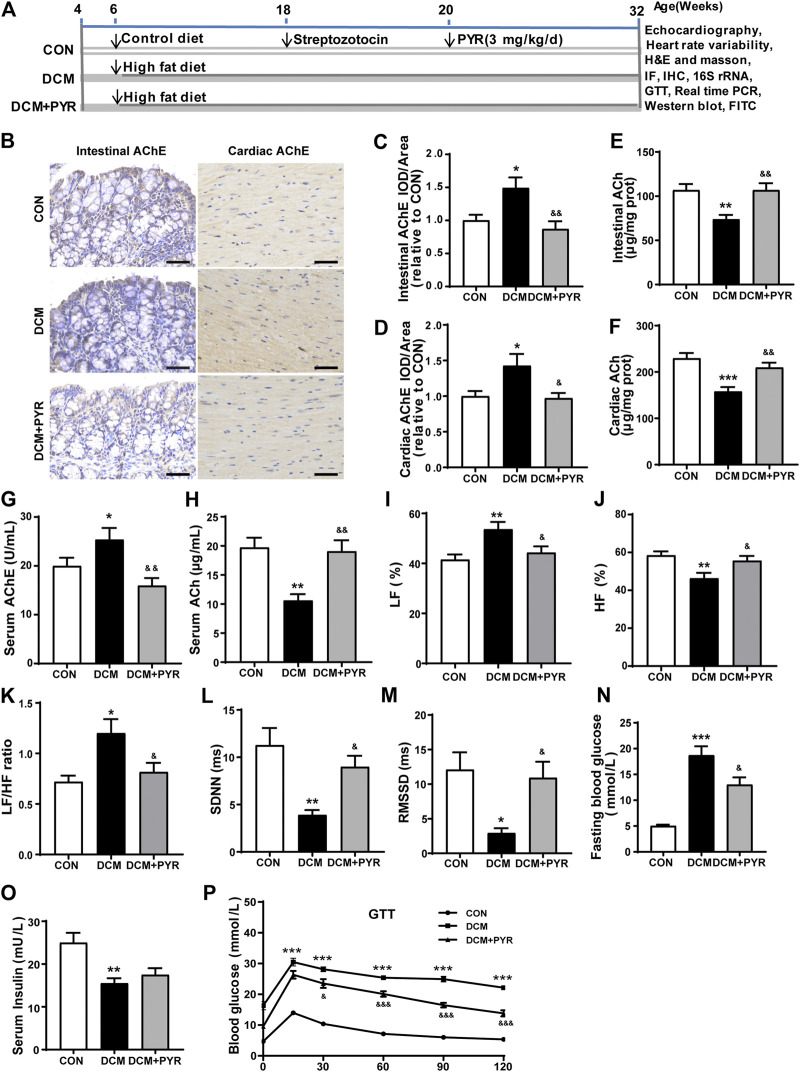
Vagal activity was decreased in diabetic cardiomyopathy mice, and pyridostigmine improved vagal activity and insulin resistance. **(A)** Experimental protocol. **(B–D)** Immunohistochemical analysis of AChE expression in intestinal and cardiac tissues; scale bar = 50 μm. **(E,F)** Intestinal and cardiac ACh concentrations. **(G,H)** Serum AChE activity and ACh concentration. **(I–K)** LF, HF, and LF/HF ratio. **(L,M)** SDNN and RMSSD. **(N)** Fasting blood glucose concentrations. **(O)** Serum insulin levels. **(P)** GTT. Data are expressed as the mean ± SEM. *n* = 6–8. *p < 0.05, **p < 0.01, ***p < 0.01, vs. CON; ^&^ *p* < 0.05, ^&&^ *p* < 0.01, ^&&&^ *p* < 0.001, vs. DCM.

After acclimatization for 2 weeks, the mice were initially administered either a normal chow diet (ND; 10% kcal fat, 70% carbohydrates, and 20% protein; D12450, Research Diets, United States) or a high-fat diet (HFD; 59.9% kcal fat, 20.1% carbohydrates, and 20% protein; D12492, Research Diets, United States) for 12 weeks. The HFD-fed mice were then intraperitoneally injected with 100 mg/kg body weight streptozocin (STZ; Sigma-Aldrich, St. Louis, MO, United States). The ND mice received equivalent volumes of 0.1 M citrate buffer. For the next 2 weeks, the mice were continually fed either the HFD or ND as before. Serum glucose levels were measured by tail blood glucometry (Roche, Basel, Switzerland) 2 weeks after the injection. Mice with fasting blood glucose levels ≥7.9 mmol/l were considered diabetic mice and were recruited for subsequent experiments (Batchu et al., 2018). Both diabetic and control mice were continually fed either the HFD or ND for 12 weeks. Then, some of the diabetic mice were intragastrically administered pyridostigmine (3 mg/kg/d) for 12 weeks (Shanghai Zhongxi Sunve Pharmaceutical Co. Ltd., Shanghai, China) (Xue et al., 2019). Accordingly, three groups were defined: the control mice + vehicle (CON) group, the diabetic cardiomyopathy mice + vehicle (DCM) group, and the diabetic cardiomyopathy mice + pyridostigmine (DCM + PYR) group (Figure 1A).

### Echocardiographic Measurement

Cardiac morphology and function were assessed by transthoracic echocardiography (VisualSonics Vevo 2100, VisualSonics, Inc., Toronto, ON, Canada) in mice anesthetized with 1.5–2% isoflurane. The left ventricular ejection fraction (LVEF), left ventricular fractional shortening (LVFS), and left ventricular internal dimensions in systole and diastole (LVIDs and LVIDd, respectively) were evaluated.

### Heart Rate Variability Analysis

HRV was calculated as the mean difference between sequential RRs for the complete set of electrocardiogram (ECG) waveforms. ECG was performed using a PowerLab system. For each 5-min stream of ECG waveforms, the mean time between successive QRS complex peaks, mean heart rate, and mean HRV analysis-generated time and frequency measures were acquired. The frequency-domain measures included low frequency (LF), high frequency (HF), and LF/HF ratio. The LF component is a marker of sympathetic modulation and the HF component is a marker of vagal modulation. The LF/HF ratio was determined as an estimate of the relative balance between sympathetic and vagal activity. The time-domain measures included the standard deviation of the normal-to-normal beat interval (SDNN) and the root mean square of successive differences (RMSSD) (Stratford et al., 2018).

### Glucose Tolerance Test

Mice were fasted overnight, and GTTs were performed after intraperitoneal injection of 2 g/kg glucose (Sigma-Aldrich). Glucose levels were measured at 0, 30, 60, 90, and 120 min using an Accu-Chek glucometer (Roche).

### *In Vivo* Intestinal Paracellular Permeability Assay

Intestinal paracellular permeability was assessed using 4 kDa fluorescein isothiocyanate–dextran (FITC-D4; Sigma-Aldrich) as a paracellular tracer. Before the assay, the mice were fasted for 6 h. The mice were then orally gavaged with FITC-D4 (500 mg/kg of body weight). Two hours after gavage, blood was collected and serum was prepared for fluorescence detection (excitation, 490 nm; emission, 520 nm) (Cheng et al., 2018).

### Blood, Feces, and Tissue Collection and Biochemical Analysis

At end of the experiments, fecal samples were collected and stored at −80°C. After the mice were anesthetized, blood samples were obtained from the abdominal aorta, and intestinal and cardiac tissues were removed. Lipopolysaccharide (LPS), ACh, and acetylcholinesterase (AChE) were detected using a biochemical detection system (AU2700, Olympus, Melville, NY, United States). Serum insulin and BCAA levels and cardiac BCAA levels were measured using commercial enzyme-linked immunosorbent assay (ELISA) kits (Abcam, Cambridge, United Kingdom) with the manufacturer’s standards and protocols.

### Hematoxylin and Eosin and Masson’s Trichrome Staining

Mouse cardiac tissues were fixed in formalin and embedded in paraffin for sectioning into 5 μm thick sections. The sections were stained with H and E and Masson’s trichrome (Heart Biological Technology Co., Ltd., Xi’an, China) and analyzed for morphological changes. The myocyte–cardiomyocyte area and fibrosis area were measured using Image-Pro Plus 6.0 (Media Cybernetics, Silver Spring, MD, United States).

### Transmission Electron Microscopy

Mouse intestinal and cardiac tissues were fixed with 2.5% glutaraldehyde in 0.1 M phosphate buffer for 2 h at 4°C. The samples were post fixed with 1% osmium tetroxide, dehydrated in a graded ethanol series, embedded in epoxy resin, and then cut into ultrathin sections. After counterstaining with uranyl acetate and lead citrate, the sections were examined by TEM (H-7650, Hitachi, Tokyo, Japan).

### Immunohistochemistry

For immunohistochemical analysis, sections were deparaffinized through xylene and ethanol series. All sections were boiled in 10 mmol/l sodium citrate antigen retrieval buffer at 95°C for 20 min and then washed three times with PBS. The sections were exposed to 3% hydrogen peroxide for 15 min to quench endogenous peroxidase activity and then washed three times with PBS. Next, the sections were blocked with 10% goat serum for 1 h and then incubated overnight at 4°C with anti-Bax (1:200 dilution; Bioworld, Minnesota, United States), anti–Bcl-2 (1:200 dilution; Bioworld), anti-cleaved caspase 3 (1:200 dilution; Bioworld), anti-zonula occludens-1 (ZO-1; 1:150 dilution; Bioworld), anti-occludin (1:200 dilution; Abcam), anti-claudin 1 (1:200 dilution; Abcam), and anti-AChE (1:200 dilution; Proteintech, Wuhan, China) antibodies. After three washes with PBS, the sections were incubated with secondary antibodies for 30 min at 37°C and then washed three times with PBS. Diaminobenzidine was used to develop the antibody staining, and hematoxylin counterstaining was used to visualize the nuclei. Images were obtained under a light microscope (BX53, Olympus, Japan) and analyzed using Image-Pro Plus 6.0 (Media Cybernetics).

### Immunofluorescence

Paraffin-embedded samples were prepared according to standard histological procedures. Nitrotyrosine (1:200 dilution; Santa Cruz, CA, United States) was used for immunostaining. The sections were then incubated with a goat anti-Mouse IgG superclonal secondary antibody for 1 h. Fluorescence images were analyzed using Image-Pro Plus 6.0 (Media Cybernetics).

### Western Blotting

Mouse cardiac tissue proteins were extracted with protease inhibitor–containing lysis buffer. The sample proteins were resolved by SDS-PAGE and transferred to polyvinylidene fluoride membranes. Nonspecific binding to the membranes was blocked by incubating the membranes in Tris-buffered saline containing 5% nonfat milk and 0.1% Tween 20 at room temperature for 1 h. The immunoblots were probed with antibodies against branched-chain amino transferase 2 (BCAT2; 1:2000 dilution; Proteintech), branched-chain α–keto acid dehydrogenase (BCKD) kinase (BCKDK; 1:1000 dilution; Proteintech), mitochondrial phosphatase 2C (PP2Cm; 1:1000 dilution; Proteintech), branched-chain α–keto acid dehydrogenase (BCKDHA; 1:1000 dilution; Bioworld), p-BCKDHA (Ser293; 1:1000 dilution; Thermo Fisher, Massachusetts, United States), and GAPDH (1:5000 dilution; CMCTAG, Milwaukee, WI, United States) overnight at 4°C and then incubated with the corresponding secondary antibodies at room temperature for 1 h. The bands were visualized with ECL-Plus reagent (Millipore) and quantified using ImageJ software (National Institutes of Health, Bethesda, MD, United States).

### High-Throughput Sequencing

Genomic DNA was extracted using an E.Z.N.A. Soil DNA Kit (Omega Bio-tek, Norcross, GA, United States) according to the manufacturer’s protocols. The concentrations and purity of the resultant DNA were determined using a NanoDrop ND-2000 (NanoDrop, United States), and the quality was assessed by running aliquots on gels. The samples were stored at –80°C for further analysis.

The 16S rRNA gene was amplified by polymerase chain reaction (PCR) with the primers 341F (5′-ACT​CCT​ACG​GGA​GGC​AGC​AG-3′) and 806R (5′-GGACTACHVGGGTWTCTAAT-3′), which target the hypervariable V3–V4 region of the bacterial 16S rRNA gene. PCR was performed in triplicate with Phusion High-Fidelity PCR Master Mix (New England Biolabs) using 30 ng of template DNA. The PCR products were purified with AMPure XP beads and quantified/qualified with an Agilent 2100 Bioanalyzer (Agilent, CA, United States). PCR products from different samples were mixed equally and used to construct an Illumina paired-end library with a NEBNext Ultra™ DNA Library Prep Kit for Illumina (NE, United States). Then, the amplicon library was sequenced in paired-end mode (2 × 300 bp) on an Illumina MiSeq platform (Illumina, San Diego, CA, United States) according to standard protocols.

### RNA Extraction and Real-Time PCR

Total RNA was isolated and extracted from cardiac tissue using TRIzol Universal (BioTeke, Beijing, China) according to the manufacturer’s protocol. The extracted RNA was quantified and assessed for integrity using a NanoDrop ND-2000 (Thermo Fisher). A kit (BioTeke) was used to perform first strand cDNA synthesis according to the procedure recommended by the manufacturer. Real-time PCR was performed on an Exicycler 96 PCR detection system (Bioneer, Daejeon, Korea). β-actin served as a control. The real-time PCR primer sequences are shown in Table 1. The relative mRNA expression levels of individual genes were calculated after normalization to the corresponding β-actin mRNA levels.

**TABLE 1:** Primer sequences in real-time PCR.

Gene	Forward primer sequence (5’ - 3’)	Reverse primer sequence (5’ – 3’)
BCKD	AGG​GCC​GGA​TCT​CCT​TCT​ACA​T	CCT​TGC​CTG​GGT​CAT​TCA​CG
BCAT2	GTC​ATC​TTG​CCT​GGA​GTA​GTT​CG	TTG​CTT​GCC​TTC​ATA​CAG​GAT​TT
PPM1K	GCC​AGG​TGT​TCT​CGG​TTT​GA	TGG​TTT​GCC​GTA​CTT​GAT​GC
BCKDK	CGT​AGC​CTT​CCT​TTC​ATC​ATT​G	CCT​CAT​CTG​CCT​GGT​CCT​TG
β-Actin	CTG​TGC​CCA​TCT​ACG​AGG​GCT​AT	TTT​GAT​GTC​ACG​CAC​GAT​TTC​C

### Statistical Analysis

The data are expressed as the mean ± SEM. The data were statistically analyzed using one-way ANOVA followed by Tukey’s multiple comparison test (three groups). Student’s t test was applied for comparison of two groups. All figures were prepared using GraphPad Prism 7.04 (GraphPad Software Inc., La Jolla, CA).

## Results

### Vagal Activity Was Decreased in Diabetic Cardiomyopathy Mice, and Pyridostigmine Improved Vagal Activity and Insulin Resistance

In the present study, a diabetic cardiomyopathy mouse model was established using a HFD and STZ. Compared with the CON group, the DCM group exhibited higher AChE activity and lower ACh concentrations in intestinal and cardiac tissues (Figures 1B–F). In addition, serum AChE activity was higher and ACh concentrations were lower in the DCM group than in the CON group (Figures 1G,H). Compared with those in the CON group, the HF was lower and the LF and LF/HF were higher in the DCM group (Figures 1I–K). The SDNN and RMSSD values in the HRV time domain were lower in the DCM group than in the CON group (Figures 1L–M). However, pyridostigmine decreased AChE activity in serum, intestinal, and cardiac tissues; increased ACh concentrations in serum, intestinal, and cardiac tissues; augmented HF, SDNN, and RMSSD values; and reduced LF and LF/HF ratio (Figures 1A–M). Together, these data suggested that autonomic imbalance was characterized by increased cardiac sympathetic tone and decreased vagal tone in diabetic cardiomyopathy mice, and that these changes were partially prevented by pyridostigmine.

Fasting serum glucose was increased and serum insulin levels were decreased in the DCM group than in the CON group (Figures 1N,O). Moreover, the GTT results revealed diminished glucose tolerance under diabetic conditions, indicating metabolic disorder in diabetic cardiomyopathy mice (Figure 1P). Fasting serum glucose levels were decreased but serum insulin levels were not notably augmented in the DCM + PYR group than in the DCM group (Figures 1N,O). Glucose tolerance was partially restored in the DCM + PYR group (Figure 1P).

### The Intestinal Barrier Was Damaged in Diabetic Cardiomyopathy Mice and Attenuated by Pyridostigmine

**FIGURE 2:**
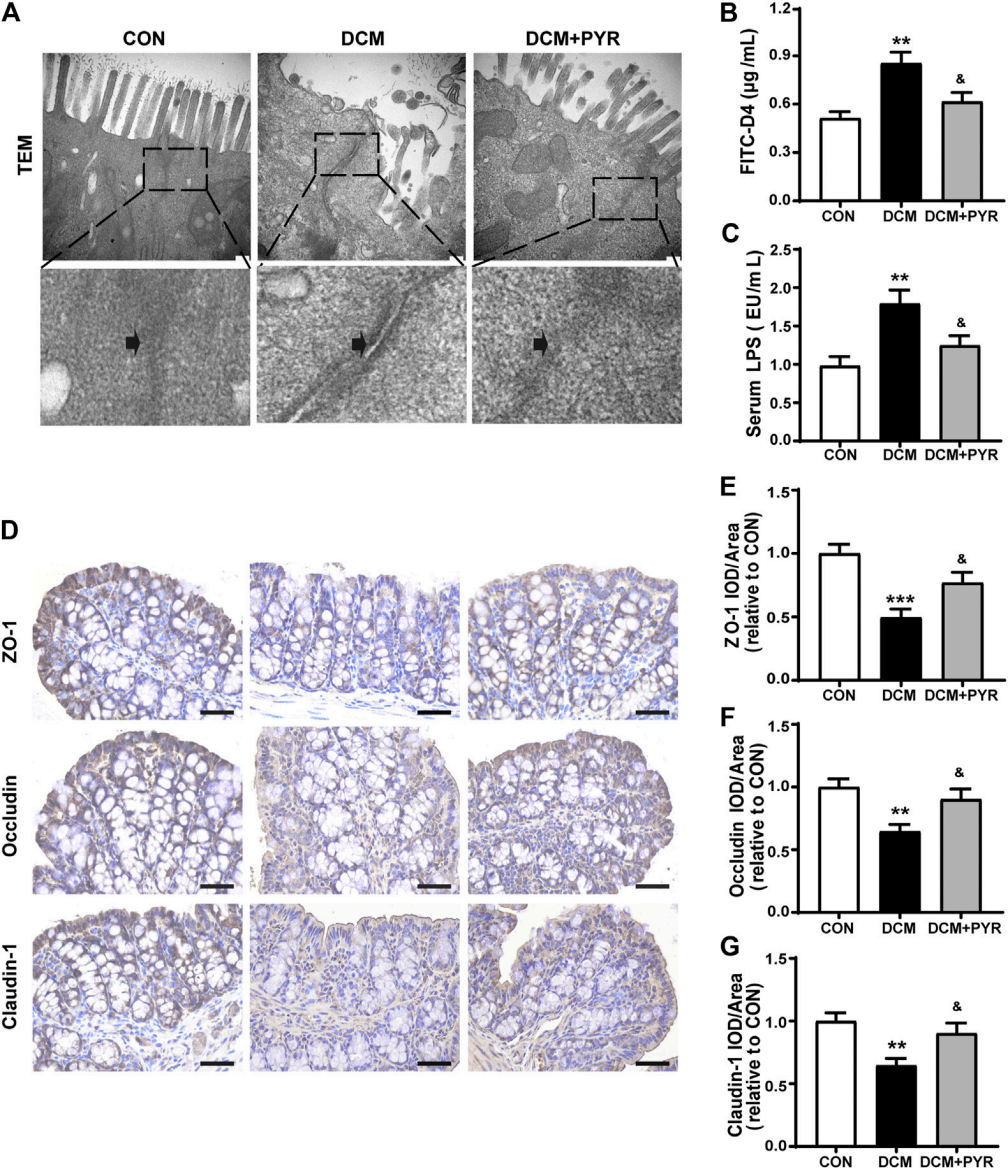
The intestinal barrier was damaged in diabetic cardiomyopathy mice and attenuated by pyridostigmine. **(A)** TEM analysis of the TJs ultrastructure of intestinal tissue; scale bar = 200 nm. **(B)** Serum FITC-D4 concentration. **(C)** Serum LPS concentration. **(D–G)** Immunohistochemical analysis of the expression of TJ proteins (ZO-1, occludin, and claudin-1) in intestinal tissue; scale bar = 50 μm. Data are expressed as the mean ± SEM. *n* = 6–8. **p < 0.01, ***p < 0.01, vs. CON; ^&^ *p* < 0.05, vs. DCM.

The TEM results revealed that the intestinal mucosal epithelium was neatly arranged with that the tight junctions (TJs) between epithelial cells were tight in the CON group. Conversely, in the DCM group, intestinal epithelial cells were swollen, and TJs between epithelial cells appeared damaged with widened intercellular spaces (Figure 2A). Intestinal permeability was measured *via* determination of the concentration of serum FITC-D4. Serum FITC-D4 levels were higher in the DCM group than in the CON group (Figure 2B). In addition, the concentrations of the serum metabolic endotoxemia marker LPS were increased in the DCM group compared with the CON group (Figure 2C). TJs are important components of the intestinal barrier, and ZO-1, occludin, and claudin-1 are key factors. The results demonstrated that ZO-1, occludin, and claudin-1 expressions were decreased in the intestinal tissues of diabetic cardiomyopathy mice (Figures 2D–G).

The TEM results also showed that TJs were distributed in an orderly manner and that widening of intercellular spaces was mild after pyridostigmine treatment (Figure 2A). The serum FITC-D4 and LPS levels were lower in the DCM + PYR group than in the DCM group (Figures 2B,C). In addition, the expressions levels of ZO-1, occludin, and claudin-1 in intestinal tissues were increased after pyridostigmine administration (Figures 2D–G).

### Pyridostigmine Restructured Gut Microbiota Homeostasis in Diabetic Cardiomyopathy Mice

**FIGURE 3:**
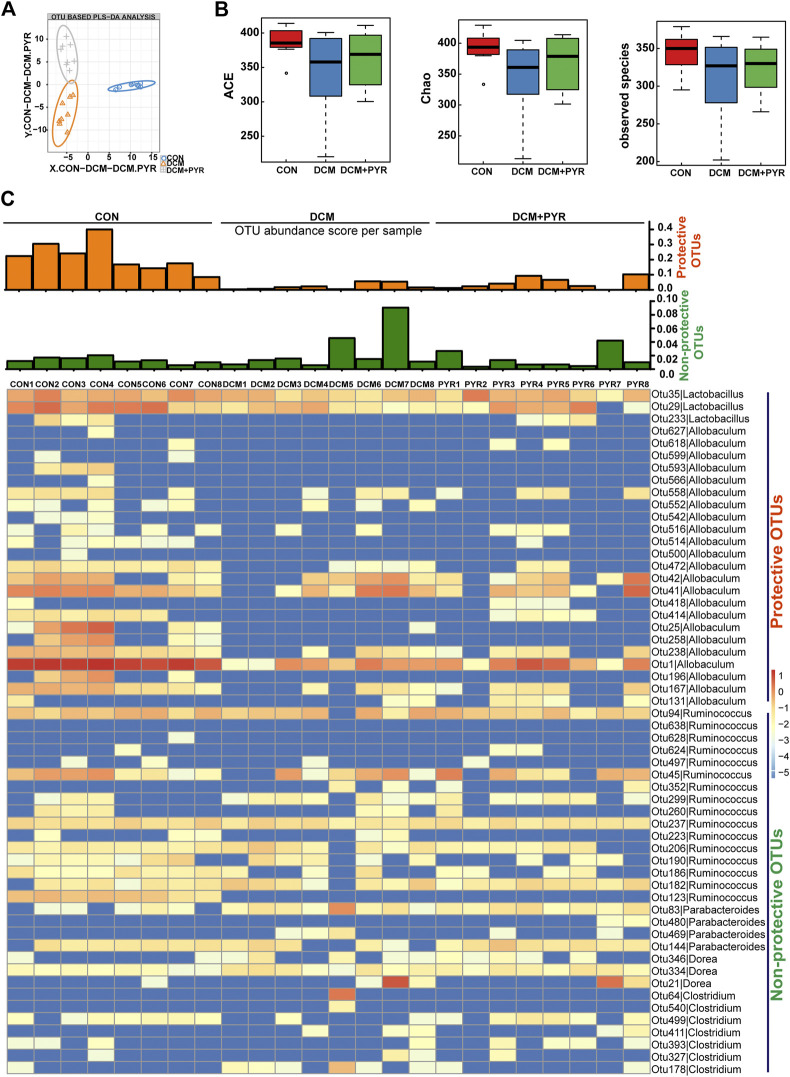
Pyridostigmine restructured gut microbiota homeostasis in diabetic cardiomyopathy mice. **(A)** PLS-DA of gut microbes based on the OTU data of the groups. **(B)** α-diversity indices (ACE, Chao, and observed species) from fecal sample 16 S rDNA sequencing data. **(C)** Heatmap of differentially abundant OTUs among CON, DCM, and DCM + PYR groups. The rows present the 56 OTUs identified in mouse fecal samples. The columns depict the mice in CON, DCM, and DCM + PYR groups. The bar graphs above the heatmap present the abundance scores of potentially protective (orange) and non-protective (green) OTUs calculated for each mouse group. *n* = 8.

Gut microbes are critical for the intestinal epithelial barrier function and physiological homeostasis maintenance (Dejea et al., 2018). Sequencing of the V3–V4 region of the 16S rRNA gene was performed on fecal samples. Partial least squares discriminant analysis (PLS-DA) of bacterial operational taxonomic units (OTUs) of the three groups showed separation of the populations (Figure 3A). Interestingly, the gut microbial taxonomy was different among the CON, DCM, and DCM + PYR groups. The α-diversities of the gut microbiota were analyzed using the ACE and Chao, and observed species indices revealed discrepancies in microbial species among the three groups (Figure 3B). The gut microbiota diversity was lower in diabetic cardiomyopathy mice than in control mice, but the decrease in diversity was attenuated by treatment with pyridostigmine.

Analysis of fecal samples identified 56 OTUs that were differentially abundant among the CON, DCM, and DCM + PYR groups. To present the data in aggregate, we counted the potentially protective (more abundant in the CON group than in the DCM group; *n* = 26) and potentially non-protective (more abundant in the DCM group than in the CON group; *n* = 30) OTUs. In addition, we calculated a score by weighing each OTU based on its relative abundance in the sample (hereafter called the abundance score) (Figure 3C). As shown in the heatmap, the abundance of protective OTUs (*Lactobacillus* and *Allobaculum*) was lower, while the abundance of non-protective OTUs (*Ruminococcus*, *Parabacteroides*, *Dorea*, and *Clostridium*) was higher in the DCM group than in the CON group. Pyridostigmine increased the abundance of protective OTUs and decreased the abundance of non-protective OTUs (Figure 3C).

### Gut Microbiota Composition Was Affected by Pyridostigmine Supplementation in Diabetic Cardiomyopathy Mice

**FIGURE 4:**
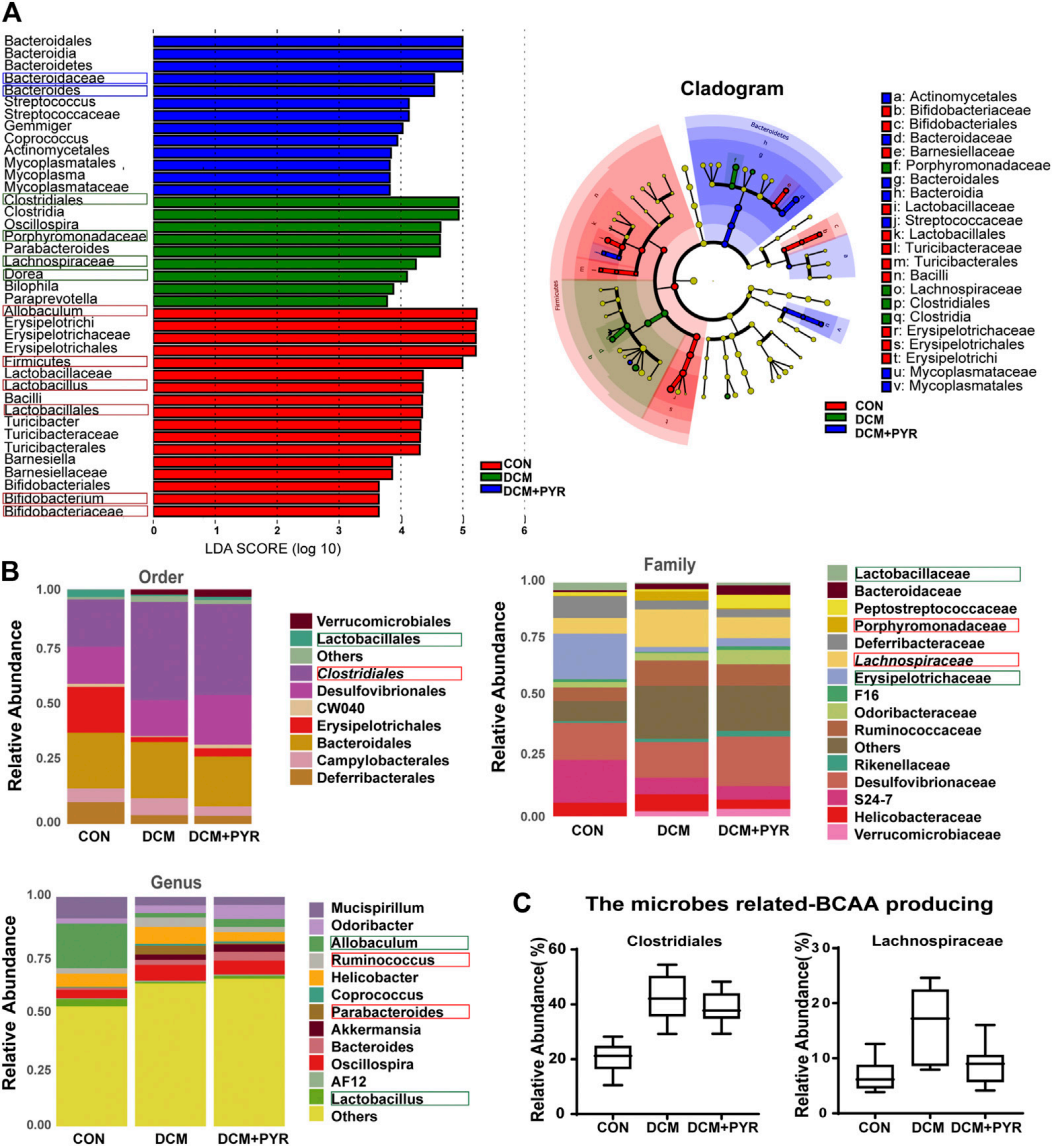
Gut microbiota composition was affected after pyridostigmine supplementation in diabetic cardiomyopathy mice. **(A)** Differences in the bacterial communities obtained by LEfSe analysis of the 16 S sequences. **(B)** Order-level, family-level, and genus-level taxonomic distributions of the gut microbiota. **(C)** Effects of pyridostigmine treatment on the relative abundance (%) of gut microbes related to BCAA production, including *Clostridiales* and *Lachnospiraceae*. Data are expressed as the mean ± SEM. *n* = 8.

To identify the specific bacterial taxa associated with diabetes and affected by pyridostigmine, we compared the gut microbiota of control mice, diabetic cardiomyopathy mice, and pyridostigmine-supplemented mice using the linear discriminant analysis (LDA) effect size (LEfSe) method. A cladogram showing the structure of the gut microbiota and the predominant bacteria was created, and the greatest differences in taxa among the control mice, diabetic cardiomyopathy mice, and pyridostigmine-supplemented mice are displayed. In the DCM group, the relative abundance levels of *Firmicutes*, *Lactobacillales*, *Lactobacillus*, *Bifidobacteriaceae*, *Bifidobacterium*, and *Allobaculum* were decreased, whereas the relative abundance levels of *Clostridiales*, *Porphyromonadaceae*, *Lachnospiraceae*, and *Dorea* were increased. Pyridostigmine supplementation significantly increased the relative abundance levels of Bacteroidaceae and *Bacteroides* but reduced the relative abundance levels of *Clostridiales*, *Porphyromonadaceae*, *Lachnospiraceae*, and *Dorea*. The differential abundance of these taxa among groups was further supported by LEfSe analysis (Figure 4A). As shown in Figure 4B, the order-level analysis demonstrated that the abundance of *Lactobacillales* was decreased, while that of *Clostridiales* was increased in the DCM group; pyridostigmine partially reversed these changes. Compared to control mice, diabetic cardiomyopathy mice displayed significantly lower relative abundance levels of *Lactobacillaceae* and *Erysipelotrichaceae* and higher relative abundance levels of *Porphyromonadaceae* and *Lachnospiraceae*, whereas pyridostigmine treatment protected against these changes. At the genus level, diabetic cardiomyopathy mice displayed significantly lower relative abundance levels of *Allobaculum* and *Lactobacillus* and higher relative abundance levels of *Ruminococcus* and *Parabacteroides*, whereas pyridostigmine treatment reversed these effects (Figure 4B).

The relative abundance levels of the bacterial taxa analyzed above not only are related to the pathological state of diabetes but also may be related to the synthesis of BCAAs. Analysis of the microbes related to BCAA production showed that the relative abundance levels of *Clostridiales* and *Lachnospiraceae* were increased in diabetic cardiomyopathy mice than in control mice, but the relative abundance levels of *Clostridiales* and *Lachnospiraceae* decreased after administration of pyridostigmine (Figure 4C).

### Pyridostigmine Improved BCAA Catabolism in Cardiac Tissue in Diabetic Cardiomyopathy Mice

**FIGURE 5:**
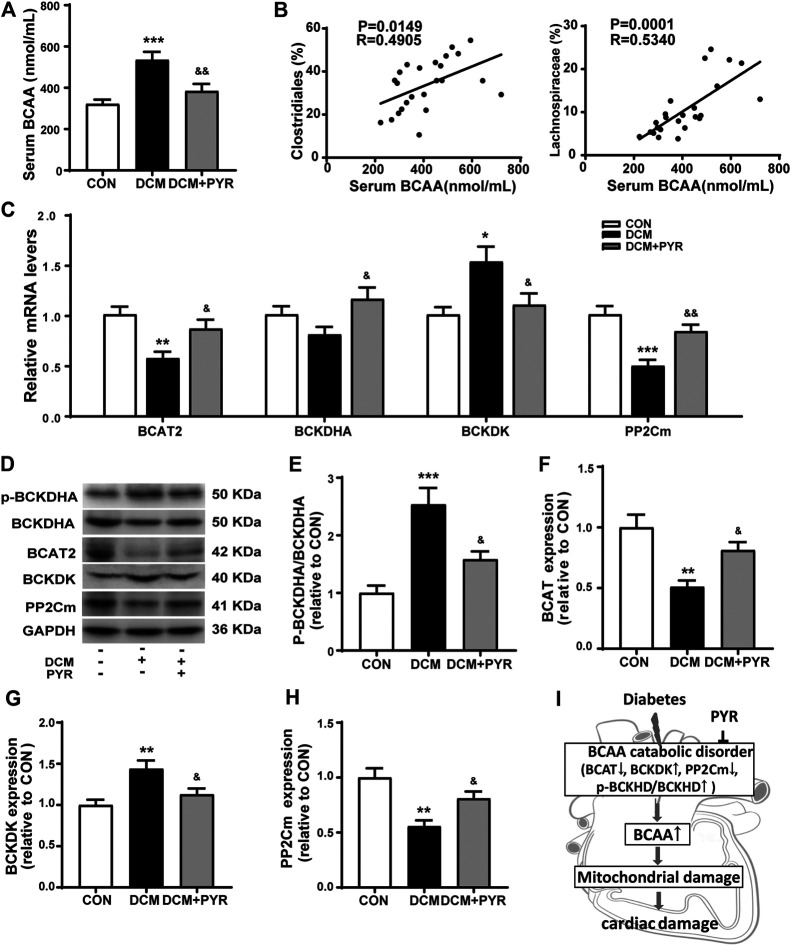
Pyridostigmine improved BCAA catabolism in cardiac tissue in diabetic cardiomyopathy mice. **(A)** Serum BCAA concentrations. **(B)** Correlation analysis between the relative abundance of gut bacteria, including Clostridiales and Lachnospiraceae, and serum BCAA levels. **(C)** Real-time PCR analysis of the mRNA expression of BCAT2, BCKDHA, BCKDK, and PP2Cm in cardiac tissues. **(D–H)** Protein expression of p-BCKDHA, BCKDHA, BCAT2, BCKDK, and PP2Cm in cardiac tissue. **(I)** The BCAA catabolism pathway in cardiac tissue was impaired in diabetic cardiomyopathy mice. Data are expressed as the mean ± SEM. *n* = 6–8. *p < 0.05, **p < 0.01, ***p < 0.01, vs. CON; ^&^ *p* < 0.05, ^&&^ *p* < 0.01, vs. DCM.

Interestingly, the relative abundance of gut microbes related to BCAA production was increased in the DCM group but pyridostigmine reversed this increase. To further determine whether changes in the gut microbiota increased circulating BCAA concentrations, serum BCAA concentrations were measured. Serum BCAA concentrations were significantly increased in diabetic cardiomyopathy mice than in control mice, but the increase was reversed by pyridostigmine administration (Figure 5A). Additionally, Spearman’s correlation analysis showed that the relative abundance levels of *Clostridiales* and *Lachnospiraceae* were positively correlated with the BCAA concentrations in serum (Figure 5B).

To investigate the effects of pyridostigmine on BCAA catabolism, the levels of the catabolic enzymes BCAT2, p-BCKDHA, BCKDHA, BCKDK, and PP2Cm were investigated by western blot analysis and real-time PCR, respectively. The real-time PCR results showed that BCAT2 and PP2Cm mRNA levels were lower and that BCKDK mRNA levels were higher in diabetic cardiomyopathy mice than in control mice. These changes were partially normalized by pyridostigmine administration. BCKDHA mRNA levels were not significantly altered in diabetic cardiomyopathy mice but were increased by pyridostigmine (Figure 5C). In addition, BCKDHA phosphorylation and BCKDK protein expression were increased, whereas BCAT2 and PP2Cm protein expression was decreased in diabetic cardiomyopathy mice. However, all of the alterations in these parameters observed in diabetic cardiomyopathy mice were partially relieved by pyridostigmine treatment (Figures 5D–H). Taken together, these results showed that cardiac BCAA catabolism was reduced in cardiac tissue in the context of diabetic cardiomyopathy but that pyridostigmine improved BCAA catabolism (Figure 5I).

### Pyridostigmine Decreased Cardiac BCAA Concentrations to Attenuate Cardiac Damage in Diabetic Mice

**FIGURE 6:**
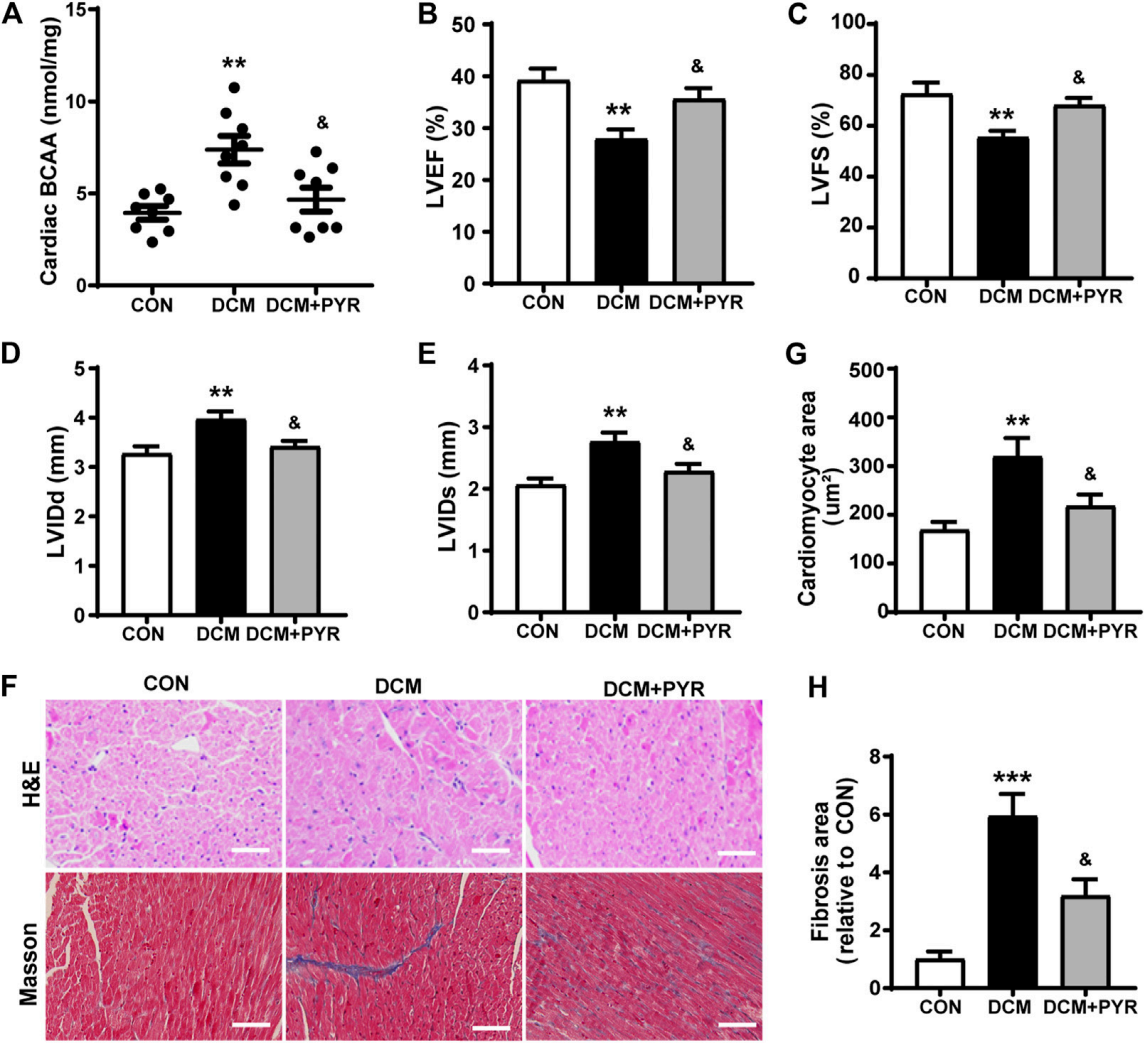
Pyridostigmine decreased cardiac BCAA concentrations to attenuate cardiac damage in diabetic mice. **(A)** BCAA concentrations in cardiac tissue. **(B–E)** LVEF, LVFS, LVIDs, and LVIDd. **(F–H)** Representative images and quantification of H&E and Masson’s staining of cardiac tissue; scale bar = 50 μm. Data are expressed as the mean ± SEM. *n* = 6–8. **p < 0.01, ***p < 0.01, vs. CON; ^&^ *p* < 0.05, vs. DCM.

Pharmacological promotion of systemic BCAA catabolism reduces circulating and cardiac BCAA concentrations and improves cardiac function during both hemodynamic and ischemic challenges (Neinast et al., 2019). Our results showed that the BCAA concentrations in cardiac tissue were increased in diabetic cardiomyopathy mice than in control mice. This abnormality in diabetic cardiomyopathy mice was greatly attenuated by pyridostigmine administration (Figure 6A).

The mice in the DCM group exhibited decreased LVEF and LVFS and increased LVIDs and LVIDd than the CON group. Pyridostigmine administration led to improvements in LVEF, LVFS, LVIDs, and LVIDd (Figures 6B–E). The cardiomyocyte and fibrotic areas were increased in the DCM group than in the CON group but were reduced by pyridostigmine administration (Figures 6F–H).

### Pyridostigmine Alleviated Cardiac Mitochondrial Dysfunction in Diabetic Cardiomyopathy Mice

**FIGURE 7:**
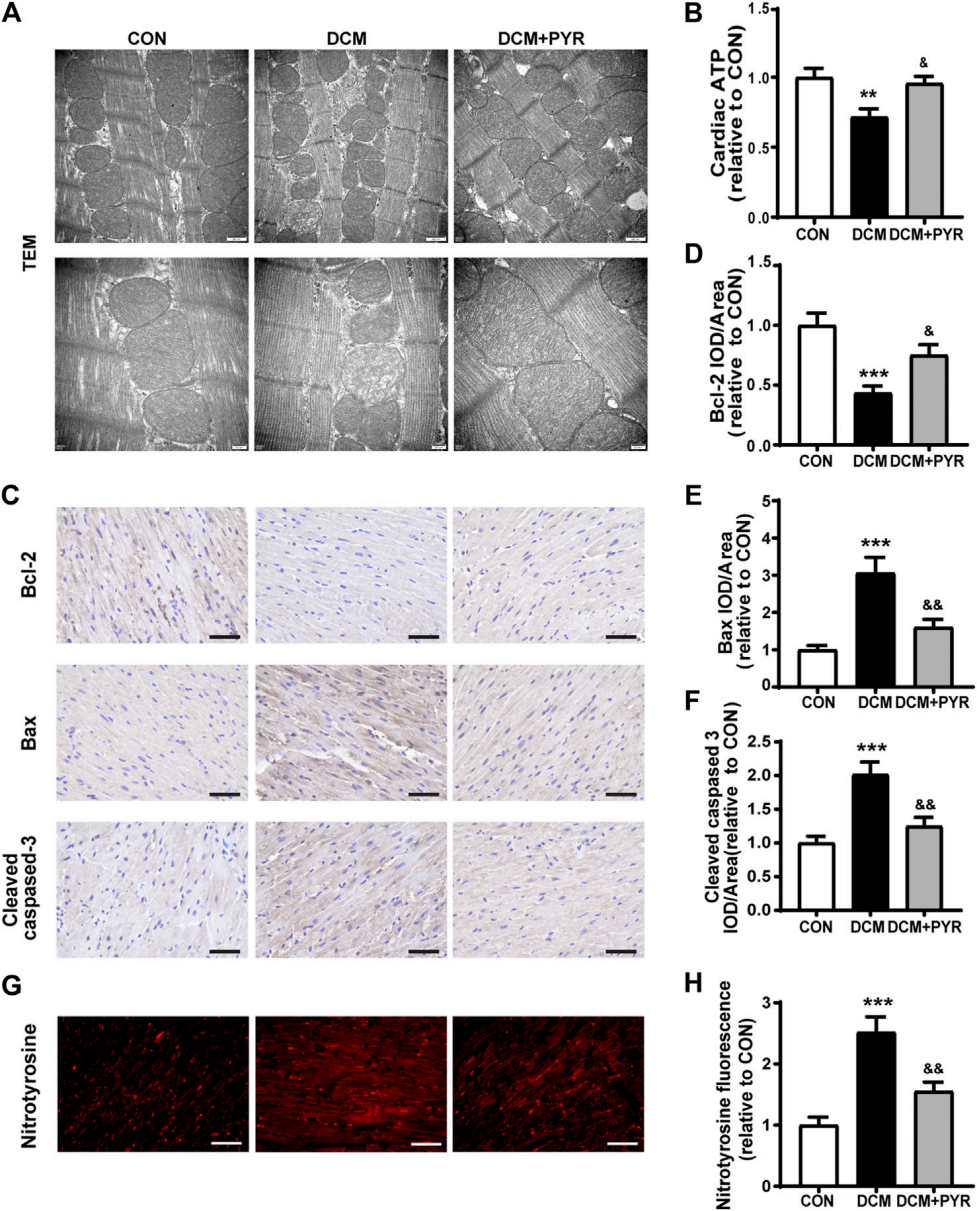
Pyridostigmine alleviated cardiac mitochondrial dysfunction in diabetic cardiomyopathy mice. **(A)** Representative transmission electron micrographs of mitochondria in cardiac tissues; scale bar = 500 nm **(top)**, 200 nm **(bottom)**. **(B)** ATP levels in cardiac tissues. **(C–F)** Immunohistochemical analysis of Bax, Bcl-2, and cleaved caspase-3 expression in cardiac tissue; scale bar = 50 μm. **(G–H)** Immunohistochemical analysis of nitrotyrosine expression in cardiac tissue; scale bar = 50 μm. Data are expressed as the mean ± SEM. n = 6–8. **p < 0.01, ***p < 0.01, vs. CON; ^&^ *p* < 0.05, ^&&^ *p* < 0.01, vs. DCM.

To further verify how BCAAs improved cardiac function in diabetic cardiomyopathy mice with pyridostigmine administration, we examined mitochondrial function in cardiac tissue. The DCM group exhibited a disordered arrangement of myocardial mitochondria, as indicated by increased proliferation and swelling, increased numbers of vacuoles, a loosened and broken mitochondrial ridge structure, and decreased matrix density. These changes were reversed by pyridostigmine (Figure 7A). ATP levels were decreased in the DCM group compared with the CON group but increased in the DCM + PYR group (Figure 7B).

Compared with control mice, diabetic cardiomyopathy mice exhibited reduced cardiac Bcl-2 expression and increased Bax and cleaved caspase-3 expression, whereas pyridostigmine treatment ameliorated these alterations and reversed mitochondria-related apoptosis (Figures 7C–F). In addition, immunofluorescence staining revealed that nitrotyrosine expression was increased in the cardiac tissue of diabetic cardiomyopathy mice. However, the expression of nitrotyrosine was significantly reduced in the DCM + PYR group (Figures 7G,H). These abnormalities were greatly alleviated by pyridostigmine administration, demonstrating that pyridostigmine improved mitochondrial function in diabetic cardiomyopathy mice.

## Discussion

The gut microbiota and its metabolite BCAAs are closely associated with metabolic syndrome and cardiovascular health conditions, including obesity, diabetes, atherosclerosis, hypertension, and heart failure (Tang and Hazen, 2014; Santisteban et al., 2017). Therefore, novel pharmacological agents to treat disruption of the gut microbiota and BCAA metabolism in diabetes are urgently needed. This study showed that diabetes resulted in autonomic imbalance and cardiac damage that was associated with disorder of the gut microbiota and BCAA catabolism. Importantly, pyridostigmine enhanced vagal activation and exerted beneficial effects on insulin resistance, mitochondrial dysfunction, and cardiac damage in the context of diabetes. To our knowledge, this is the first report of the protective effect of pyridostigmine against intestinal barrier injury, gut microbiota disruption, and BCAA catabolism disruption. This study provides novel insights for the development of a therapeutic strategy for diabetes-induced cardiac damage that targets the gut microbiota and BCAA catabolism.

Patients with diabetes have chronic hyperglycemia, which commonly leads to tissue damage and dysfunction of the heart, blood vessels, and other organs (Zhang et al., 2016). Diabetic cardiomyopathy, a major cardiovascular complication in diabetic patients and is defined as structural and functional myocardial impairment without coronary artery disease or hypertension, is characterized mainly by myocardial hypertrophy and fibrosis, metabolic dysregulation, and defects in myocardial contractile properties (Cosentino et al., 2020). In the current study, a diabetic cardiomyopathy mouse model was established via HFD feeding and STZ administration. Several studies have used HFD feeding to induce obesity, insulin resistance, and diabetes in rodents and large animal (Sorop et al., 2018). A previous study has shown that feeding mice HFD with 60% fat content leads to cardiac systolic dysfunction (Battiprolu et al., 2012). Numerous studies have subjected animals to HFD feeding combined with STZ treatment to induce β-cell dysfunction and insulinopenia, which are long-term symptoms of diabetes (Mali et al., 2014; Nath et al., 2017; Barrière et al., 2018). Similar to HFD feeding, additional treatment with STZ promoted oxidative stress, cardiac hypertrophy, and contractile dysfunction in diabetic mice (Mali et al., 2014; Nath et al., 2017). Consistently, the diabetic cardiomyopathy model mice in the present study exhibited elevated fasting serum glucose levels, left ventricular dysfunction, myocardial hypertrophy, and fibrosis.

Previous studies have demonstrated that autonomic imbalance, as indicated by attenuated parasympathetic nerve tone and increased sympathetic nerve activity, is involved in the pathological processes of diabetes (Coats and Cruickshank, 2014). Pyridostigmine was first used for treating patients with myasthenia gravis and has been repeatedly tested in the clinic (Lorenzoni et al., 2020). Recently, some studies have revealed that pyridostigmine activates the vagal nerve to elicit favorable effects on myocardial ischemia, myocardial infarction, and heart failure in both animal and clinical experiments (Androne et al., 2003; Lataro et al., 2013). Our previous studies showed that pyridostigmine increased ACh levels by inhibiting cholinesterase, increasing HRV and baroreflex sensitivity, and improving cardiac function in animal models with cardiovascular diseases without significantly affecting on the cardiac function of normal rats (Lu et al., 2017; Lu et al., 2018; Xue et al., 2019). The data presented here provide the first direct evidence that vagal discharge was reduced in the gut tissues of diabetic cardiomyopathy model. The results showed that pyridostigmine decreased AChE activity, increased ACh levels in serum and in gut and cardiac tissues, and exerted beneficial effects on insulin resistance and cardiac damage in diabetic cardiomyopathy mice.

Intestinal barrier dysfunction and gut microbiota disruption have been linked to various diseases, including atherosclerosis, hypertension, heart failure, obesity, and diabetes (Tang and Hazen, 2014; Santisteban et al., 2017). Intestinal barrier dysfunction may participate in diabetes mellitus development by increasing intestinal permeability, thus triggering an inflammatory response leading to peripheral insulin resistance and ultimately to diabetes mellitus (Gong et al., 2017; Sorini et al., 2019). Hyperglycemia-mediated barrier disruption facilitated harmful bacterial antigens and microorganisms from the intestinal lumen to blood, lymph, and extraintestinal tissues (Thaiss et al., 2018; Allam-Ndoul et al., 2020). Normally, the intestinal epithelium prevents pro-inflammatory LPS translocation, but circulating LPS levels are elevated in diet-induced obese and diabetic mice (Clemente-Postigo et al., 2019). Research revealed that LPS induced marked cardiac dysfunction, apoptosis, and inflammation in myocardial injury models (Xin et al., 2021). Interestingly, vagal nerve electrical stimulation potently reduces intestinal inflammation by restoring intestinal homeostasis, whereas vagotomy has the reverse effect (Matteoli and Boeckxstaens, 2013). Consistently, the TEM results in the current study showed that the TJs between epithelial cells appeared damaged with widened intercellular spaces in diabetic cardiomyopathy mice. In addition, TJ protein expression was decreased, while intestinal permeability (indicated by LPS, FITC-D4 in serum) was increased in diabetic cardiomyopathy mice. Pyridostigmine alleviated the abnormal TJ ultrastructure of intestinal tissue, increased TJ protein expression, and decreased intestinal permeability.

Gut microbes are critical for intestinal epithelial barrier function and for the maintenance of physiological homeostasis (Dejea et al., 2018; Ma and Li, 2018). Previous studies have demonstrated that the α-diversity of the gut microbiota is associated with obesity and diabetes (Cao et al., 2019). This study showed that the α-diversity index in diabetic cardiomyopathy mice was decreased, but this effect was reversed by pyridostigmine. Accumulating studies have reported that diabetic patients and animals exhibit significantly different gut microbiota compositions compared with their nondiabetic counterparts (Wang and Jia, 2016). For example, Larsen et al. reported that the proportion of bacteria in the phylum *Firmicutes* was significantly lower in a diabetic adult male group than in a healthy control group (Larsen et al., 2010). Hu et al. found that the abundance of *Erysipelotrichaceae* was reduced in prediabetic mice and therefore proposed that mice with reduced *Erysipelotrichaceae* abundance may subsequently develop diabetes (Hu et al., 2018). Qin et al. reported that the abundance of *Lachnospiraceae* bacteria in the gut was positively correlated with type 2 diabetes, implying that *Lachnospiraceae* might be associated with the occurrence of diabetes (Qin et al., 2012). Gu et al. demonstrated that *Porphyromonadaceae* was positively correlated with fasting insulin and fasting blood glucose in HFD mice (Gu et al., 2020). Furthermore, *Bifidobacterium*, *Lactobacillus*, and *Bacteroides* are beneficial bacteria that can directly affect the immune system of the host, inducing intestinal immunity and enhancing immune function (Yuan et al., 2018). Additionally, accumulating studies have demonstrated that the abundance levels of *Ruminococcus*, *Parabacteroides*, *Dorea*, *Clostridiales*, and *Clostridium* are increased, whereas those of *Lactobacillales*, *Lactobacillaceae*, *Lactobacillus*, *Allobaculum*, *Bacteroides*, *Bifidobacterium*, and *Bifidobacteriaceae* are decreased in diabetic animal models and patients (Peng et al., 2014; Gil-Cardoso et al., 2016; Cui et al., 2019; Wang et al., 2019; Zhang et al., 2019; Li S. et al., 2020; Ma et al., 2020). In this study, pyridostigmine enhanced the abundance of *Lactobacillales*, *Lactobacillaceae*, *Erysipelotrichaceae*, *Lactobacillus*, and *Allobaculum*. In contrast, pyridostigmine reduced the abundance of *Clostridiales*, *Porphyromonadaceae*, *Lachnospiraceae*, *Ruminococcus*, *Parabacteroides*, *Dorea*, and *Clostridium*. Notably, *Firmicutes*, *Lactobacillales*, *Bifidobacteriaceae*, *Bacteroidaceae*, *Allobaculum*, *Lactobacillus*, *Bifidobacterium*, and *Bacteroides* were the predominant bacterial groups in control and pyridostigmine treatment groups. The predominant bacterial groups in the diabetic cardiomyopathy model group were *Clostridiales*, *Porphyromonadaceae*, *Lachnospiraceae*, and *Dorea*.

Disturbance of the intestinal flora affects functional metabolites of the intestinal microbiota, such as BCAAs, short-chain fatty acids, and trimethylamine N-oxide. Recent research has demonstrated that elevated BCAA levels are associated with gut microbiome patterns characterized by enriched BCAA biosynthetic potential (Newgard et al., 2009). In addition, the relative abundance of BCAA-producing *Clostridiales* bacteria is increased in HFD-fed mice (Yue et al., 2019). The family *Lachnospiraceae* is positively associated with circulating BCAAs in different European populations (Ottosson et al., 2018). In the current study, pyridostigmine decreased the abundance of BCAA-biosynthesizing microbes, including *Clostridiales* and *Lachnospiraceae*. Epidemiological research has demonstrated that serum BCAA levels are elevated in insulin-resistant individuals and that these elevations are correlated with elevated abundance of BCAA-producing microbes (Pedersen et al., 2016). Consistently, our study showed that circulating BCAA concentrations were higher in diabetic cardiomyopathy mice than in control mice. Treatment with pyridostigmine decreased the circulating BCAA concentrations partially. In addition, Pearson correlation analysis indicated that the abundance levels of *Clostridiales* and *Lachnospiraceae* were positively correlated with the serum concentrations of BCAAs.

Normally, surplus BCAAs in the circulatory system can be catabolized via abundant BCAA catabolizing enzymes in various tissues (Zhou et al., 2019). The BCKD complex (BCKDC) is a multienzyme complex that exists in mitochondria and is regulated by kinases (BCKD phosphorylation inactivation) and phosphatases (BCKD dephosphorylation activation). In the context of diabetes, BCAA catabolism in skeletal muscle tissue was impaired, leading to accumulation of high BCAA levels (Lian et al., 2020). Previous research showed that cardiac BCAA catabolism was impaired in the context of human heart failure but that enhancing BCAA oxidation could improve the function of the failing heart (Uddin et al., 2019). Suppression of whole-body BCAA catabolism via deletion of PP2Cm elevates circulating and cardiac BCAA levels and worsens the cardiac response to ischemia/reperfusion injury (Li et al., 2017). In addition, a previous study revealed that increased plasma BCAA levels were associated with long-term adverse cardiovascular events in patients with ST-segment elevation myocardial infarction and acute heart failure (Du et al., 2018). Pharmacological promotion of systemic BCAA catabolism reduces circulating and cardiac BCAA levels and improves cardiac function during both hemodynamic and ischemic challenges (Wang et al., 2016). Consistently, the present study showed that BCAA catabolism was downregulated in cardiac tissues of diabetic cardiomyopathy mice and was changed by pyridostigmine.

Mitochondrial dysfunction has been identified as a mechanism related to cardiometabolic diseases and underlying cardiovascular risk factors, such as diabetes (Xu et al., 2015). Mitochondrial damage is also accompanied by the development of inflammation (Jia et al., 2018). The coordinated activation of inflammatory signals by BCAAs may interfere with mitochondrial biosynthesis and energy metabolism, and mitochondrial dysfunction and inflammation can further lead to BCAAs accumulation (Ye et al., 2020). Previous study showed that high concentrations of BCAAs augment oxidative damage and mitochondrial dysfunction in human peripheral blood mononuclear cells (Zhenyukh et al., 2017). Lian et al. provided evidence that lack of PP2Cm-induced oxidative stress and mitochondrial morphological changes, such as mitochondrial swelling (Lian et al., 2015). In addition, high levels of BCAA metabolites resulted in mitochondrial dysfunction and apoptosis in obese Zucker rats that exhibited BCKDC impairments (Olson et al., 2014). This study showed that pyridostigmine ameliorated mitochondrial structure disorder and dysfunction, mitochondria-related oxidative stress, and apoptosis in cardiac tissues in the context of diabetic cardiomyopathy.

## Conclusion

**FIGURE 8:**
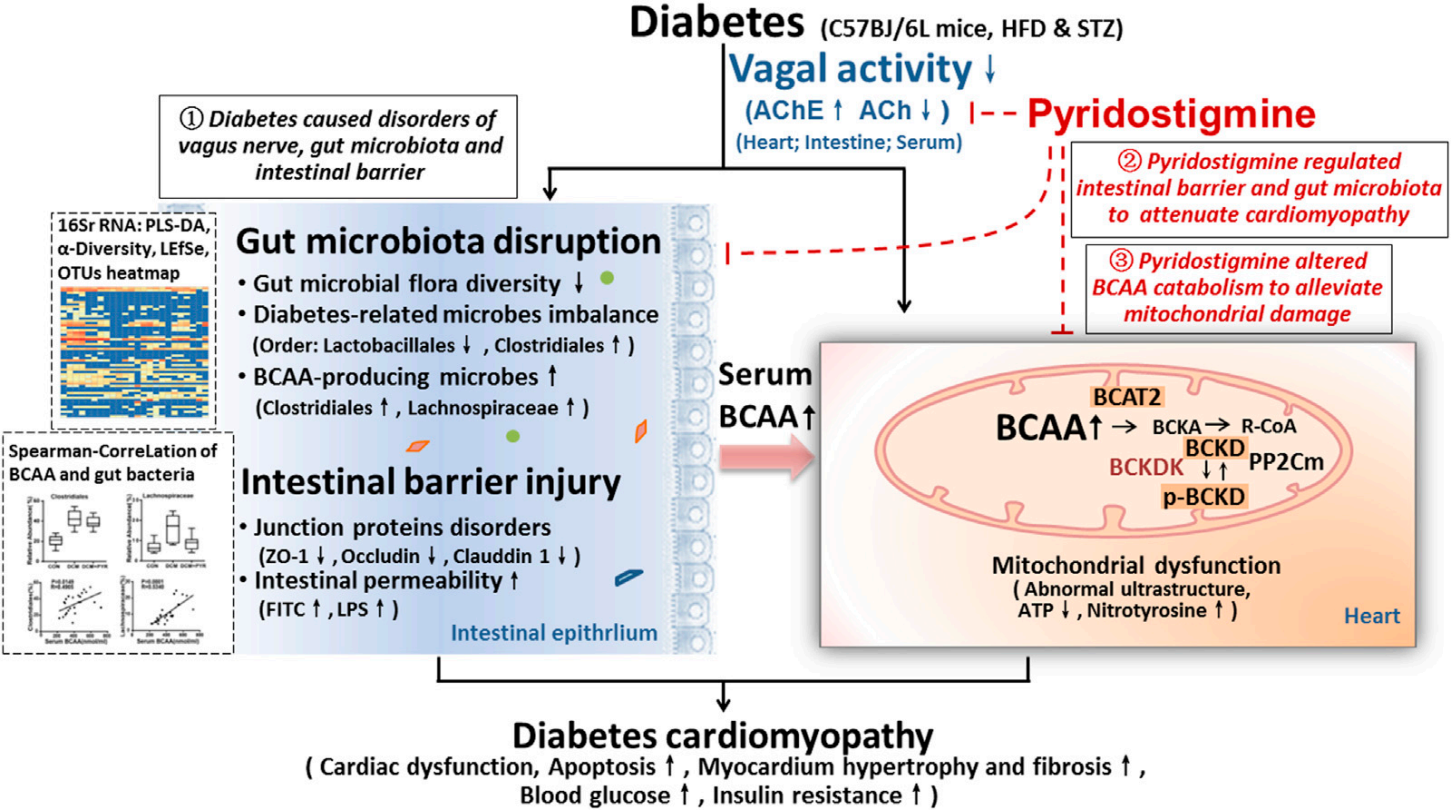
Illustration of the effect of pyridostigmine on diabetic cardiomyopathy and the proposed mechanism. (1) Intestinal barrier function (related to TJs and intestinal permeability) and gut microbial homeostasis (diabetes-related and BCAA-producing microbes) were impaired in diabetic cardiomyopathy mice, and this impairment was accompanied by reduced vagal activity, which eventually led to cardiac damage. (2) Pyridostigmine enhanced vagal activity and alleviated intestinal barrier injury and gut microbiota disruption, thereby reducing BCAA levels and ameliorating cardiac damage. (3) More importantly, BCAA catabolism was decreased in cardiac tissue in the context of diabetes, whereas pyridostigmine regulated BCAA catabolism enzymes to decrease cardiac BCAA concentrations and alleviate mitochondrial dysfunction.

The present study demonstrated the following: 1) Intestinal barrier function (related to TJs and intestinal permeability) and homeostasis of gut microbes (diabetes-related and BCAA-producing microbes) were impaired in diabetic cardiomyopathy mice, and this impairment was accompanied by reduced vagal activity, which eventually led to cardiac damage. 2) Pyridostigmine enhanced vagal activity and alleviated intestinal barrier injury and gut microbiota disruption, thereby reducing BCAA levels and ameliorating cardiac damage. 3) More importantly, BCAA catabolism was decreased in cardiac tissue in the context of diabetes, but pyridostigmine regulated BCAA catabolism enzymes to decrease cardiac BCAA concentrations and alleviate mitochondrial dysfunction. Taken together, these findings showed that pyridostigmine enhanced vagal activity, attenuated intestinal barrier injury and gut microbial disruption, and improved BCAA catabolism to attenuate cardiac damage in diabetic cardiomyopathy mice (Figure 8). Overall, our study provides evidence for the roles of the gut microbiota and BCAA catabolism in diabetes-induced cardiac damage and contributes novel insights for the development of therapeutic strategies related to vagal activation.

## Data Availability

The datasets presented in this study can be found in online repositories. The names of the repository/repositories and accession number(s) can be found below: BioProject: PRJNA691099.
